# Disordered Eating Behaviors Through the Lens of Self-Determination Theory

**DOI:** 10.5964/ejop.v14i3.1577

**Published:** 2018-08-31

**Authors:** Catherine Bégin, Annie Fecteau, Marilou Côté, Alexandra Bédard, Caroline Senécal, Carole Ratté

**Affiliations:** a School of Psychology, Laval University, Quebec, Canada; b Institute of nutrition and functional foods, Laval University, Quebec, Canada; c Faculty of Medicine, Laval University, Quebec, Canada; Department of Psychology, Webster University Geneva, Geneva, Switzerland; Maynooth University, Maynooth, Ireland

**Keywords:** self-determination theory, disordered eating, basic psychological needs, eating regulation, life satisfaction

## Abstract

This study aimed to verify a conceptual model of eating regulation based on the Self-Determination Theory. This model suggests that basic psychological needs satisfaction is related to general self-determined motivation and autonomous regulation toward eating, which in turn are associated with less disordered eating behaviors and attitudes and better satisfaction with life. Two hundred thirty-nine women without an eating disorder completed self-reported questionnaires. The hypothesized model was tested with a serial multiple mediation analysis using PROCESS macro. The overall indirect effect of basic psychological needs satisfaction on life satisfaction through the three mediators, i.e. general motivation, regulation of eating behaviors, and eating behaviors and attitudes, was significant. Results are coherent with the Self-Determination Theory and add to past research by suggesting that basic psychological needs satisfaction might be a key target when addressing women’s disordered eating behaviors and attitudes.

Much effort has been devoted in last decades to prevent and reduce disordered eating behaviors. It becomes clear that a deeper understanding of the etiology of these disorders and of the motivational processes potentially involved in their development are needed to develop more effective public health initiatives and interventions (Matusitz & Martin, 2013; Verstuyf, Patrick, Vansteenkiste, & Teixeira, 2012).

A conceptual framework of human motivation which has been studied in light of disordered eating is the Self-Determination Theory (SDT) (Verstuyf et al., 2012). The SDT argues that individuals can build a self-determined motivation and develop their full potential only if the three basic psychological needs (autonomy, competency and relatedness needs) are satisfied (Deci & Ryan, 1985, 1991, 2000). According to the SDT, autonomy refers to the perception of an individual that he/she is the source of his/her own behaviors and that he/she is acting in line with his/her values and interests. Competency refers to the feeling of being effective and to the tendency to seek the opportunity to express and develop one’s own capacities. Relatedness refers to the desire to care for reciprocal self and others well-being, and the need to feel connected to others (Deci & Ryan, 2002). The satisfaction of these basic psychological needs significantly contributes to individuals’ self-determined motivation, which leads individuals to behave specifically in accordance with their own values and goals (autonomous regulation), and experiences psychological well-being. Inversely, individuals for whom basic psychological needs are not satisfied develop a less self-determined motivation in general, and are more prone to behave in response to internal and external pressures or to avoid negative consequences (controlled regulation), which is linked to detrimental effects on psychological well-being (Deci & Ryan, 2000, 2008; Ryan & Deci, 2000).

Some evidence suggests that unsatisfied basic needs or basic needs thwarting for autonomy, competence and relatedness may be implicated in the etiology of disordered eating behaviors. Needs satisfaction has been demonstrated to be related to a healthier diet (e.g. higher intake of fruits and vegetables) (Ryan, Patrick, Deci, & Williams, 2008) while needs thwarting is associated with unhealthy weight control behaviors (Thøgersen-Ntoumani, Ntoumanis, & Nikitaras, 2010) and a greater presence of eating disorder symptoms (Kopp & Zimmer-Gembeck, 2011; Schüler & Kuster, 2011). As reviewed by Verstuyf et al. (2012), general thwarting of the basic psychological needs seems to lead to need substitutes, such as endorsement of the thin ideal and body dissatisfaction, and to compensatory behaviors, such as rigid restrictive eating and/or excessive uncontrolled eating. These results suggest that control over eating behaviors, body and weight could be a way to compensate underlying autonomy, competency and relatedness basic deficiencies (Deci & Ryan, 2000).

In addition, as proposed by Pelletier and collaborators, SDT may be an interesting model to examine motivational processes underlying disordered eating behaviors (Pelletier & Dion, 2007; Pelletier, Dion, & Lévesque, 2004; Pelletier, Dion, Slovinec-D’Angelo, & Reid, 2004). Their results support that women who are more self-determined are less prone to endorse sociocultural pressures toward thinness, to be dissatisfied with their body image, and to adopt bulimic symptoms (Pelletier, Dion, & Lévesque, 2004). Moreover, general self-determination toward one’s life is a determinant of the motivation toward the regulation of eating behaviors. As well, the motivation toward the regulation of eating behaviors is an important predictor of eating behaviors per se and psychological well-being. Indeed, two other studies conducted by Pelletier and colleagues (Pelletier & Dion, 2007; Pelletier, Dion, Slovinec-D’Angelo, & Reid, 2004) showed that an autonomous regulation toward eating leads to healthy eating while controlled regulation toward eating is expected to be associated with more bulimic and depressive symptoms, as well as with a negative self-esteem and a poorer life satisfaction.

Although previous findings suggest that the SDT represents a promising theoretical framework to understand the motivational processes involved in disordered eating behaviors and attitudes, literature is still scarce and more research is needed (Verstuyf et al., 2012). Previous studies which examined eating behaviors through the lens of the SDT (Pelletier & Dion, 2007; Pelletier, Dion, & Lévesque, 2004; Pelletier, Dion, Slovinec-D’Angelo, & Reid, 2004) did not include in their explanatory model the contribution of basic psychological needs satisfaction, while needs satisfaction is the basis of this theory. It would be therefore interesting to examine whether basic needs satisfaction predicts eating behaviors and life satisfaction through a mechanism involving general motivation and motivation toward eating. Thereby, the present study aimed to verify empirically a global conceptual model of eating regulation based on the SDT. This model proposes that basic psychological needs satisfaction is related to general self-determined motivation and autonomous regulation toward eating, which in turn is associated with less disordered eating behaviors and attitudes and greater life satisfaction.

## Methods

### Participants and Procedure

Two hundred thirty-nine adult women without eating disorders were recruited in Quebec City, Canada, through email lists that reach the Laval University community (students and employees). Participants mean age was 22.8 year (*SD* = 4.3). Most of them were students (91%). Regarding their marital status, they reported being mainly single, separated or divorced (66%). Seventy percent had obtained a college degree, and 27% had obtained a university degree, 3% had a high school degree or less. Sixty-five percent were normal weight, 14% were underweight, 16% were overweight and 5% were obese.

All women interested in the study were invited to the laboratory. They first signed the informed consent form, which had been approved by the Laval University’s Institutional Review Board. Thereafter, they completed self-reported questionnaires assessing variables under study.

### Measures

Basic psychological needs satisfaction was evaluated with the Basic Psychological Needs Scale (Deci & Ryan, 2000), a 21-item scale measuring competency, autonomy and relatedness. Participants rated their satisfaction of psychological needs on a 7-point Likert scale, with higher scores reflecting a higher level of satisfaction. This instrument has a good internal consistency (Gagné, 2003). An alpha coefficient of .76 for the total scale was observed in the present study.

The Global Motivational Scale - short version (Guay, Mageau, & Vallerand, 2003) is a 16-item questionnaire with a 7-point Likert scale measuring general motivation in life. It assesses intrinsic motivation, identified, introjected and external regulation, as well as amotivation. The global self-determination index was calculated for each participant, with higher scores reflecting higher degree of self-determined motivation. This instrument has high level of reliability and validity among adult population (Vallerand, 1997). In the present sample, alpha coefficients for subscales ranged from .72 to .87.

The Regulation of Eating Behaviors Scale (Pelletier, Dion, Slovinec-D’Angelo, & Reid, 2004), a 48-item questionnaire, was used to assess reasons for which women regulate their eating behaviors on a 7-point Likert scale. Participants indicated the extent to which each item was compatible to their personal motive for regulating their eating behaviors in response to the question: “Why are you regulating your eating behaviors?”. This instrument comprise following subscales: intrinsic motivation, integrated, identified, introjected and external regulation as well as amotivation, from which a total score was derived. Higher scores indicated a higher degree of autonomous regulation of eating behaviors. In the present sample, alpha coefficients for subscales range from .73 to .88.

Disordered eating behaviors and attitudes were measured by the Eating Disorders Inventory-2 (EDI-2) (Garner, 1991), a 91-item questionnaire measuring eating pathology on a 6-point Likert scale. Only three subscales directly assessing eating behaviors and attitudes were used in this study (i.e. Bulimia, Body Dissatisfaction, and Drive for thinness), and they were combined to form a total mean score. A higher score indicated a higher degree of disordered eating behaviors and attitudes. The EDI-2 has good psychometric properties (convergent and discriminant validity) (Garner, Olmstead, & Polivy, 1983). In the present sample, alpha coefficients ranged from .65 to .92.

The Satisfaction with Life Scale (Diener, Emmons, Larsen, & Griffin, 1985) is a 5-item questionnaire assessing general satisfaction with one’s life on a 7-point Likert scale. A global score was calculated by summing the five items. A higher score indicated a higher satisfaction with life. In the present sample, alpha coefficient was .86.

### Statistical Analyses

Assumptions regarding normal distribution were verified. Analyses were performed using SPSS v24.0. Means, standard deviations and Pearson’s correlations between study variables were examined. The hypothesized model was tested with a serial multiple mediation analysis using PROCESS macro (Hayes, 2013). The independent variable was basic need satisfaction, the dependent variable was life satisfaction, and the mediators were general motivation, specific motivation in the regulation of eating behaviors as well as disordered eating behaviors and attitudes. PROCESS produces bias-corrected bootstrap samples (10,000 samples) to generate 95% confidence intervals (CIs) for the indirect effect (IE) of each mediator, as well as for the serial indirect effect of the three mediators. A significant indirect effect is found when the confidence intervals do not include zero.

Prior to analyses, all variables were z-standardized. Potential multivariate outliers were screened through Mahalanobis distance. Two cases were identified as multivariate outliers (*p* < .001). Since there was no significant difference in the mediation results when the two outliers were included, the analyses include these outliers. Two participants were dropped from analyses because of missing data on regulation of eating behaviors. The analysed sample consisted of 237 participants.

## Results

### Descriptive and Correlation Analyses

Table 1 shows means, standard deviations, and Pearson’s correlation coefficients for study variables. In general, there were moderate to strong relationships between variables included in the model, as proposed by the SDT.

**Table 1:** Means, Standard Deviations, and Pearson’s Correlation Coefficients for Study Variables

Variable	*M*	*SD*	1	2	3	4	5
1. Basic needs satisfaction	113.03	15.22	-	.600**	.423**	-.311**	.618**
2. Global motivation	9.07	3.87		-	.412**	-.190**	.402**
3. Regulation of eating behaviors	19.06	9.17			-	-.268**	.314**
4. Disordered eating behaviors and attitudes	3.88	3.75				-	-.399**
5. Satisfaction with life	27.57	5.57					-

*Note. N* = 237.

**р < .01.

### Serial Multiple Mediation Analysis

**Figure 1:**
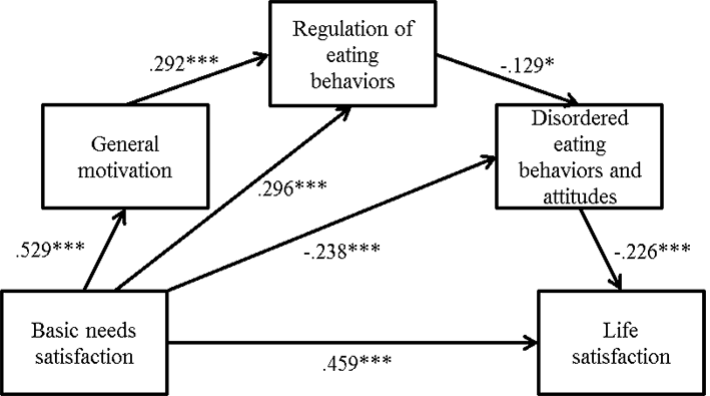
Serial multiple mediation analysis relating basic psychological needs satisfaction, general motivation, regulation of eating behaviors, disordered eating behaviors and attitudes, and life satisfaction. *Note.* *N* = 237. Values are standardized regression coefficient; only significant paths are reported. (The exact same model has been reproduced with only the disorder eating attitudes). *p < .05. **p < .01. ***p < .001.

The serial mediation model’s results first showed a significant direct effect of basic psychological needs satisfaction on life satisfaction (β = .1912, *SE* = .058; *p* < .0001). The overall indirect effect of basic needs satisfaction on life satisfaction through the three mediators, i.e. general motivation, regulation of eating behaviors, and disordered eating behaviors and attitudes, was also significant (IE = .0045, 95% bootstrap CI .001 to .014). The specific indirect effects of basic needs satisfaction on life satisfaction through (1) regulation of eating behaviors, and disordered eating behaviors and attitudes (IE = .009, 95% bootstrap CI .001 to .029), as well as only through (2) disordered eating behaviors and attitudes (IE = .054, 95% bootstrap CI .020 to .110) were also both significant. No other specific indirect effects were found to be significant (CIs included zero). Figure 1 shows significant paths (*p* < .05) and their regression coefficients.

## Discussion

The aim of the present study was to get a better understanding of the possible etiological factors of disordered eating behaviors and attitudes, and their underlying motivational processes, by testing the links between basic psychological needs satisfaction, global motivation, motivation to regulate eating behaviors, disordered eating behaviors and attitudes, and life satisfaction. A model based on the SDT and inspired by Pelletier and his colleagues’ work (Pelletier & Dion, 2007; Pelletier, Dion, & Lévesque, 2004; Pelletier, Dion, Slovinec-D’Angelo, & Reid, 2004) was elaborated and tested among women from the general population. The tested model succeeded in bringing out a significant serial mediation effect, showing that the more women’s basic psychological needs are satisfied, the more their motivation is self-determined, the more they regulate their eating behaviors autonomously, the less they adopt disordered eating behaviors and attitudes, and the more they report satisfaction with their life. In line with previous studies (Pelletier & Dion, 2007; Pelletier, Dion, & Lévesque, 2004; Pelletier, Dion, Slovinec-D’Angelo, & Reid, 2004; Verstuyf et al., 2012), these findings provide a strong support to the usefulness of the SDT when studying disordered eating behaviors and attitudes and their impact on wellbeing. Although the present results are based on a general sample of women without an eating disorder, they are, nevertheless, in line with the eating disorder recovery literature, which suggests that patients who entered treatment with higher level of motivation to change, especially the intrinsic level of motivation, or who have a higher motivation during treatment, had favorable outcomes (Clausen et al., 2013; Vall & Wade, 2015).

The main contribution of the present study is the inclusion of basic psychological needs satisfaction as a precursor variable in the explanatory model of disordered eating behaviors and life satisfaction through the lens of SDT. Our findings thus support the assumption that a self-determined motivation that leads to a more autonomous regulation of eating behaviors and less disorder eating behaviors and attitudes stems from fulfilled basic psychological needs. One possible explanation could be that unsatisfied psychological needs could influence eating behaviors and attitudes through an impact on negative affect (Verstuyf, Vansteenkiste, Soenens, Boone, & Mouratidis, 2013). Since unsatisfied psychological needs are linked to negative affect (Ryan, Bernstein, & Brown, 2010), and that negative affect may serve as a trigger for pathological eating behaviors (Leehr et al., 2015), the presence of negative affect when psychological needs are not satisfied may at least partly account for the association between psychological needs satisfaction and disordered eating symptoms. This hypothesis awaits additional examination. From a clinical standpoint, it is also possible to think that women with unsatisfied basic psychological needs may try to compensate these deficiencies by building a false sense of mastering, autonomy and competency over food, something that they can control more easily than their deeper sense of being weak, powerless, and alone (Bruch, 1981; Slade, 1982).

Even though this study gives empirical support to the proposed model based on the SDT, some limitations need to be highlighted. First, associations found between variables do not demonstrate any causal link, since the cross-sectional design does not allow any inference about causality. Longitudinal studies are needed to evaluate the impact of changes across time in psychological needs satisfaction and motivational processes on disordered eating behaviors and attitudes. Second, data were collected from volunteered women who showed an interest in participating in the study. These women might have been more motivated or more self-determined in comparison to the women who did not volunteered to participate in the study and this may have influenced results when testing the underlying motivational processes toward eating behaviors and attitudes. In addition, since a majority of women was normal weight in our study and that higher BMI has been associated with lower level of intrinsic motivation and autonomous regulation, results can not be extrapolated to overweight/obese individuals (Gropper et al., 2014; Leong et al., 2012). Since we recruited women with no formal eating disorder diagnosis, we also cannot extrapolate these results to the eating disorder population. Third, constructs were mainly tested with self-reported measures, so results could have been influenced by social desirability. Therefore, conclusions should be drawn carefully.

In sum, results from this study suggest that a global conceptual model based on the SDT including basic psychological needs satisfaction may help to understand possible etiological factors implicated in disordered eating behaviors. Findings propose that satisfaction of basic psychological needs is related to self-determined motivation in general, autonomous regulation of eating and less disordered eating behaviors and attitudes, which are linked to a better life satisfaction. These results may have interesting implications from a clinical standpoint. That is, interventions on disordered eating behaviors may benefit from working on the improvement of basic psychological needs satisfaction per se, i.e. autonomy, competency and relatedness, rather than focusing only on the enhancement of self-determined motivation to quit pathological eating or promote health behaviors (Bellg, 2003). Thus, strategies promoting autonomy, and reinvestment in different spheres of the patient’s life instead of an over-investment in control over food and weight should be recommended. Future studies should examine our model among a diagnosed eating disorders sample.
